# Changes in the relationship between Index of Concentration at the Extremes and U.S. urban greenspace: a longitudinal analysis from 2001–2019

**DOI:** 10.1057/s41599-023-02115-w

**Published:** 2023-11-23

**Authors:** J. C. Kitch, T. T. Nguyen, Q. C. Nguyen, Y. Hswen

**Affiliations:** 1 Department of Statistics, Harvard University, Cambridge, MA, USA.; 2 Department of Epidemiology and Biostatistics, University of Maryland School of Public Health, College Park, MD, USA.; 3 Department of Epidemiology and Biostatistics, Bakar Computational Health Sciences Institute, University of California San Francisco, San Francisco, CA, USA.; 4 Computational Precision Health, University of California Berkeley, Berkeley, CA, USA.

## Abstract

Urban greenspace is associated with a wide variety of human health benefits, from improved mental health to reduced violent crime and infant birth outcomes. This study investigates long-term trends in the distribution of greenspace across census tracts in the 260 U.S. cities with populations greater than 100,000 residents based on economic privilege and deprivation. Satellite-acquired Normalized Difference Vegetation Index (NDVI) images were used as a measure of greenspace, and the Index of Concentration at the Extremes (ICE) was used as a measure spatial social polarizations of deprived and priveliged populations. From 2001 to 2019, the most privileged, highest ICE quintile census tracts had both the highest mean NDVI and experienced a significantly greater increase in greenspace than the least privileged tracts, indicating that the disparity in greenspace access is widening. Public greenspace initiatives need to focus on programs that are more equitable across the spectrum of economic strata to reduce urban health disparities and address concerns of environmental justice.

## Background

Vegetation in cities can take many forms, from private lawns to public parks to street trees and grassy boulevards. Past studies have shown that greenspace reduces exposure to harmful air pollutants (Hwang et al. 2011; Nowak et al. 2006), and even small parks provide important shade and heat sinks to combat the urban heat island effect (Aram et al. 2019; Park et al. 2017). Parks provide increased opportunities for physical activity, and research has suggested the power of greenspace in promoting social cohesion and directly reducing depression and stress (Beyer et al. 2014; James et al. 2015; Jennings and Bamkole 2019; Twohig-Bennett and Jones 2018; Wendelboe-Nelson et al. 2019). Other studies have suggested links between increased urban greenness and higher birth weights (Agay-Shay et al. 2014; Dadvand et al. 2012; McEachan et al. 2016), lower violent crime rates (Kuo and Sullivan 2001; Shepley et al. 2019; Venter et al. 2022), and improved long-term health outcomes.

However, a large body of scholarly work has also shown the vast, systemic inequities in the distribution of urban vegetation. Multiple studies have demonstrated strong, positive correlations between income and tree canopy cover in urban census tracts (Casey et al. 2017; Schwarz et al. 2015). Other research has examined the relationship between the “redlining” practices of the Home Owners’ Loan Corporation (HOLC) in the 1930s and the distribution of greenspace today (Nardone et al. 2021). HOLC lending classifications were a systemically racist practice that “redlined” neighborhoods to indicate poor loan quality, often targeting areas with a high minority or immigrant population. They are still associated with present-day levels of racial segregation, poverty, and income inequality (Mitchell et al. 2018). Historically redlined neighborhoods have reduced present-day greenspace (Nardone et al. 2021), experience significantly hotter temperatures (Hoffman et al. 2020), and increased air pollution from vehicle exhaust (Nardone et al. 2020). Recent work has shown that neighborhoods with a higher concentration of non-Hispanic Black residents and residents under the poverty line have significantly less total greenspace as measured by remotely-sensed satellite imagery (NDVI; see methods) than comparable White neighborhoods (Casey et al. 2017).

These findings present serious problems for notions of equitable urban public health and environmental justice in our cities. People living below the poverty line are already more likely to have chronic conditions such as asthma, diabetes, and heart disease (Osborn et al. 2013; Winkleby et al. 1992). A landmark study found a 15-year gap in life expectancy between the richest 1% and poorest 1% of Americans (Chetty et al. 2016). Access to greenspace is inextricably linked to these problems. Inequities in urban greenspace along lines of income and race perpetuate the poor air quality, excessive ambient noise, and increased susceptibility to summer heat waves that lower income and minority neighborhoods experience. The U.S. Environmental Protection Agency (EPA) defines environmental justice as the “fair treatment of all people regardless of race, color, national origin, or income, with respect to the development, implementation, and enforcement of environmental laws, regulations, and policies” so that all can enjoy “the same degree of protection from environmental and health hazards.” (US EPA 2015). Under this framework, we can understand inequities in urban greenspace as an important issue of environmental justice.

COVID-19 further highlighted the vast racial and socioeconomic health inequities throughout the U.S. and the rest of the world. Urban greenness—vegetation—is one of the most important supports for urban public health but is also one of the most privileged resources in an increasingly dense and gray city landscape. Throughout the COVID-19 pandemic, outdoor spaces became a haven for those wanting to leave their home, exercise, and socialize without putting themselves at increased risk for contracting COVID-19 (Noszczyk et al. 2022; Pouso et al. 2021). Use of hiking trails and public parks increased greatly, with a study in Oslo, Norway finding that it increased by 291% during lockdown compared to a three year average for the period of time (Venter et al. 2020). The refuge service that outdoor spaces played during COVID-19 lockdowns reinforced their critical position in the built city matrix.

With the recent availability of high-resolution, satellite-acquired greenspace data, researchers can answer critical questions about greenspace resource availability across wide geographic areas. One recent study (Casey et al. 2017) examined change in greenspace across major metropolitan areas in the U.S. using the Index of Concentration at the Extremes (ICE) as a measure of spatial social polarizations of deprived and privileged populations and satellite-acquired Normalized Difference Vegetation Index (NDVI) as a proxy for neighborhood greenspace. However, that study only examined greenspace change from 2001 to 2010. We need to be updated with the most recent data to determine if disparities are widening or shrinking. This study examines how the relationship between satellite-acquired NDVI and ICE at the census tract level has changed from 2001 to 2019. We can then understand how disparities in greenspace access have evolved over this time period. Our analysis defines “greenspace” to include all urban vegetation, which gives us a holistic picture of greenspace distribution across a city. While recent research has investigated the present-day distribution of urban greenspace across census tracts of different levels of spatial social polarization, this study provides more useful and interpretable findings because we fix the census tract boundaries of our study. This allows us to understand how greenspace has changed in small, geographically constant units in relation to the spatial social polarization of that unit. These findings will give important feedback to policymakers and city officials, making them better equipped to deal with the challenges cities will face with a changing climate.

## Methods

### Area of interest.

We set our study area to include all U.S. incorporated cities with greater than 100,000 residents. City boundaries and census tracts were drawn according to the U.S. Census Bureau 2018 Incorporated Place and Census Tract boundaries to ensure the study was based on a consistent geographic area. All statistics (ICE, NDVI, demographics) come from these census tract boundaries. While past research has used metropolitan statistical areas with greater than 100,000 residents as the study area (Casey et al. 2017), these metropolitan areas often include the outlying suburban or rural areas around a city center. We would expect the relationship between greenspace and income in these outlying rural areas to be different than that of the urban core. For instance, vegetation might be plentiful but property values significantly lower because of the lengthy commuting time to the city center. Other studies have chosen to focus their analyses within city boundaries (Barbosa et al. 2007; Gerrish and Watkins 2018) and take advantage of the dense building and population structures that make greenspace so limited. Therefore, to obtain a more accurate picture of urban greenspace distribution and change we focused our analysis inside major city boundaries. We hypothesize that this will allow for greater differentiation between urban neighborhoods.

### U.S. Census demographic data.

Demographic data was obtained from the American Community 5-year Survey (ACS) for 2010 and 2019 using the data.census.gov API. Key statistics include population density, and household income quintile thresholds. Controlling for population density would ensure that higher income areas aren’t more green only because there are less buildings and people living there.

We then measure the economic privilege of a census tract using Massey’s Index of Concentration at the Extremes (ICE) (Massey et al. 2001). While other economic indicators, such as the Gini Coefficient and median household income, could also be used to measure neighborhood social polarization, we choose ICE because of its well-established applications and justifications in public health monitoring. It captures extremes of privilege and deprivation, allowing us to explicitly understand the relationship between spatial social polarization and greenspace access (Krieger et al., 2016). We calculate it using ACS data. The ICE for Census Tract i in city j is calculated with the equation (Massey et al. 2001):

$$ICE_{ij}=\frac{\left(A_{ij}-P_{ij}\right)}{T_{ij}}$$

where $A_{ij}$ is the number of households in census tract i that have income >80th percentile for city j. Similarly, $P_{ij}$ is the number of households in census tract i that have income under the 20th percentile threshold for city j. $T_{ij}$ is the total number of households in census tract i. ICE ranges from −1 to 1, with values closer to 1 suggesting greater privilege in an area. An ICE value for a census tract can carry more information than a median household income statistic as it helps control for disparities in cost of living across cities. We used ICE measured from 2010 geographic boundaries and demographic data for our analysis. Because census tract boundaries are redrawn every ten years, ICE measured from 2001 data would not be compatible with ICE measured from 2010 to 2019 data. 2010 ICE was chosen since it was the temporal midpoint of the study.

### NDVI satellite image acquisition.

MODIS NDVI Images at 250 m resolution were obtained from the U.S. Geographical Survey (USGS) AρρEars portal in GeoTIFF format (US Geographical Survey 2022). MODIS satellites capture NDVI images every 16 days at 250m^2^ resolution, and have been used frequently in studies of urban vegetation (Casey et al. 2017; Gascon et al. 2016; Nardone et al. 2020). Since the U.S. NDVI tends to peak in the month of July, we used the first estimate in that month. Analyzing data at peak NDVI levels allows for more noticeable differences between census tracts. As a sensitivity analysis, an annual average NDVI was computed by obtaining MODIS images and averaging NDVI values from four data points (winter: January 1st; spring: April 7th; summer: July 12th; fall: September 30th). To gain insight into how urban greenspace has changed, we collected data on these dates in 2001—the earliest year NDVI data was available—2010, and 2019. 2019 was the end of our selected study period, and 2010 was a logical midpoint of this window.

After loading the GeoTIFF raster file into R Version 4.0.4 (R Core Team 2022), we used the *exactextractr* (Baston 2020) package to perform zonal statistics on census tract shapefiles. *exactextractr* was used to calculate the mean NDVI value per census tract. Raster cells that lie on a census tract boundary are weighted by the fraction of the cell that is within the census tract in question. All other cells are given a weight of “1” or “0” depending on if they lie entirely inside or outside the census tract. Less than 10 percent of census tracts covered less than one NDVI data cell.

LANDSAT, MODIS, and Sentinel-2 satellite systems all offer images that the Normalized Difference Vegetation Index (NDVI) can be extracted from. NDVI is the go-to metric for remotely sensed vegetation data, and is used across agricultural, forestry, and urban planning applications. We select MODIS as our satellite image system of choice for consistency with prior literature (Casey et al. 2017), and because the measurements are more reliable. The daily measurements of MODIS images, which the USGS combines into 16-day averages, are more robust to the effects of cloud cover than LANDSAT images which are measured less frequently but at higher resolution.

The heterogeneity of cities across the United States proved to be a challenge in analyzing NDVI trends. Cities existed in deserts, marine forests, arid plains and many other ecoregions. It is possible that cities in desert climates would have lower overall changes in NDVI than cities in wet, marine forests where vegetation is easier to maintain. While some of these city-specific effects are already addressed by our linear mixed-effects model, we include Omernik’s Level I Ecoregions to account for the ecological differences of each city in our study (Omernik 1987). Level I ecoregions divide the continental U.S. into 12 distinctecoregions, from the “Great Plains” to the “North American Deserts” and “Wet Tropical Forests” and have been used in prior greenspace research (Casey et al. 2017).

### Statistical analyses.

We fit Linear Mixed-Effects (LME) models using the *lme4* package in R Version 4.0.4 (Bates et al. 2015). LME models are highly effective when analyzing correlated measurements from grouped data. Census tracts within each city are likely to be correlated, since they share an ecological environment, municipal policies, and cultural values about greenspace. Each city spans hundreds of census tracts, and each census tract contains three separate years of NDVI measurements. We built a single-level LME model, fitting random intercepts at the city level to allow for varying levels of baseline average greenspace. We examined percentage greenspace change from 2001 to 2019, with 2001 as our baseline measurement. The percentage change is based on the nine-year intervals of our collected data (NDVI data collected in 2001, 2010, 2019).

## Results

This study examined the relationship between census tract Index of Concentration at the Extremes (ICE) and the change in the Normalized Difference Vegetation Index (NDVI) from 2001 to 2019. The study area included 23,998 census tracts across 274 U.S. cities. Table 1 displays the results of Spearman’s correlation coefficient tests for the dependent variables used in our analysis.

Census tracts in the highest ICE quintile had a 7.85% increase in greenspace from 2001–2019 (Table 2). Compared with census tracts in the highest quintile (A), all census tracts had lower percentage gains in greenspace. However, the lowest ICE census tracts did comparatively better (6.23% increase) than the D and C quintile census tracts (5.40% and 5.89% increase respectively). Overall, the lowest (E) ICE quintile census tracts had greenspace growth 20.6% slower than the A quintile and the D quintile census tracts had greenspace growth 31.2% slower.

**Fig. 1:**
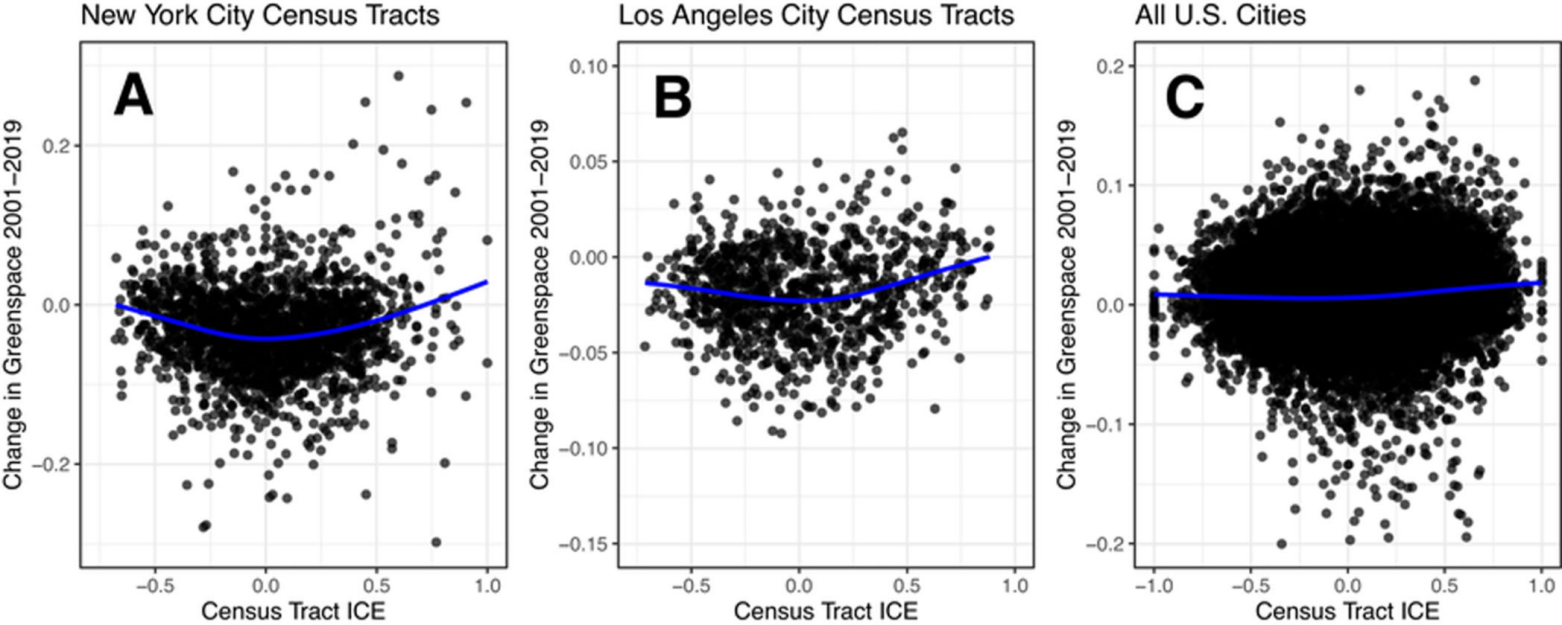
Comparison of the relationship between the change in census tract greenspace and census tract ICE. (**a**) New York City, (**b**) Los Angeles, (**c**) All U.S. Cities. Each data point represents the change in July NDVI from 2001–2019 for a single census tract, with the overall trend represented by a blue non- linear smoother.

This trend of greenspace growth follows a distinctive “J” shape illustrated in Fig. 1, where the neighborhoods with the least gains in greenspace fall within the lower-middle ICE quintile. Scatterplots of the relationship between ICE and greenspace growth in the U.S.’ two largest cities by population, New York City and Los Angeles, further demonstrates this trend.

We fit two other models to describe the relationship between greenspace growth and neighborhood ICE. Table 3 displays the results from a model where neighborhood 2010 ICE was fit as a continuous variable. A one unit increase in 2010 census tract ICE is associated with a 1.98% increase in greenspace growth. For example, the typical census tract with ICE = 0 had a 6.36% increase in mid-July NDVI from 2001 to 2019, whereas a census tract with ICE = 1 would have an NDVI increase of 8.34%.

**Fig. 2:**
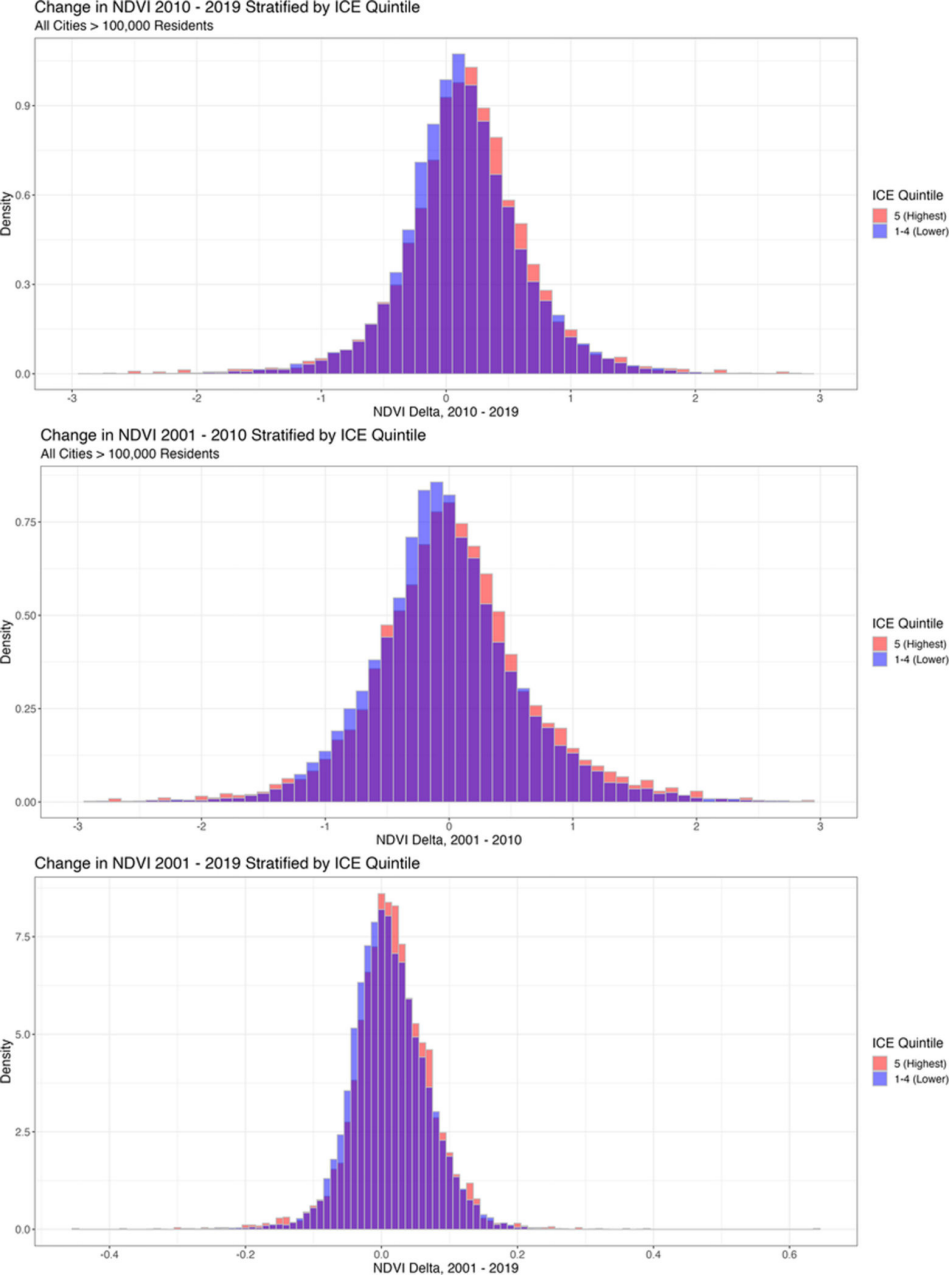
Distribution of NDVI change by ICE quintile. (**a**) Change in NDVI measured from 2001 to 2010 (2001 and 2010 satellite imagery). (**b**) Change in NDVI measured from 2010 to 2019 (2010 and 2019 satellite imagery). (**c**) Change in NDVI measured from 2001 to 2019 (2001 and 2019 satellite imagery). All ICE quintiles were measured from 2010 census tract boundaries and 2010 American Community Survey income statistics. Purple bars represent overlap between the top ICE quintile and the lower four ICE quintiles. Blue bars represent values of NDVI change where the lower four ICE quintiles have greater density, and red bars represent values where the top ICE quintile has greater density.

Table S1 displays the results of dichotomizing census tract ICE into two categories: census tracts in the highest ICE quintile (i.e., quintile “A” from Table 2)—and census tracts in the lower four B-E quintiles. The average census tract in the highest ICE quintile had a 7.85% increase in NDVI from 2001 to 2019, whereas the average census tract in the lower four ICE quintiles had a 6.01% increase in July NDVI. Figure 2 depicts the difference in the distributions of NDVI change between these two groups.

The relationship of ICE with change in NDVI was statistically significantly different from zero for all models, with a threshold of significance α <0.05. Surprisingly, the population density of a census tract was not significantly associated with the 20-year change in greenspace when controlling for ICE and ecoregion in any model.

**Fig. 3:**
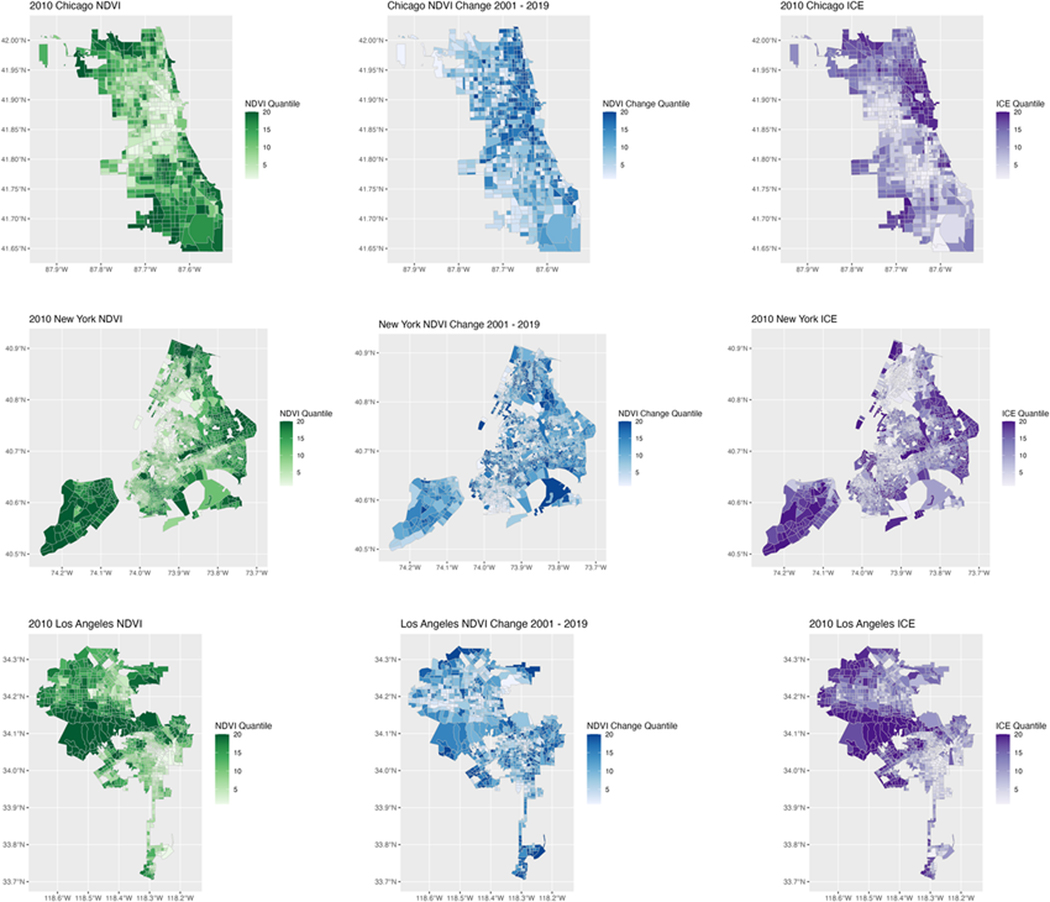
Comparison of the relationship between census tract greenspace, census tract change in greenspace, and census tract ICE. (**a**) Chicago, (**b**) New York, (**c**) Los Angeles. Darker colors represent higher NDVI (green), higher greenspace growth (blue), and higher ICE (purple). Correlations in color shade across the three panels, especially noticeable in Los Angeles and Chicago, suggest a persistence and growth of the socioeconomic gradient of urban greenspace.

Figure 3 presents a spatial representation of these findings, demonstrating the strong correlation between neighborhood 2010 ICE, 2010 NDVI, and net 2001–2019 change in NDVI across the three largest cities in the U.S. (New York City, Los Angeles, and Chicago). While only a small subset of the 260 cities included in our full analysis, we chose these cities because of their size and because we found them to be representative of the patterns in the entire study population.

## Discussion

There exist large disparities in health outcomes across different socioeconomic classes in the United States. Neighborhood greenspace is one of the many factors that can influence the health outcomes of city inhabitants. It is associated with improved air quality, better self-reported mental health, higher physical activity levels, and decreased rates of violent crime along with other impacts (Kuo and Sullivan 2001; Nowak et al. 2006; Pouso et al. 2021; Shepley et al. 2019; Wendelboe-Nelson et al. 2019). However historically redlined areas, as well as poorer areas more broadly, tend to have less greenspace than their more privileged neighbors (Casey et al. 2017; Nardone et al. 2021). Greenspace is one of the many resources attached to a higher social position in the United States. This leads to better health outcomes and a perpetuation of the socioeconomic health gradient. Therefore, a reduction in the greenspace gap between rich and poor neighborhoods could help reduce health disparities. In this study we demonstrate the persistence of disparities in urban greenspace over the last twenty years, 2001–2019. Furthermore, we uniquely suggest that these disparities may also be growing larger, even after considering seasonal changes.

This analysis demonstrated a significant, non-linear trend in the evolution of city greenspace over the course of an eighteen-year window. Most clearly, the top 20% of urban census tracts ranked by ICE Income quintile experienced the largest growth in their greenspace. This trend persists across a variety of different methods of looking at greenspace growth. It is somewhat surprising that the most privileged census tracts, which already had the most greenspace to begin with, also had the largest growth in greenspace. Since we accounted for Omernik ecoregion in our models and constructed models based on a census tract’s yearly average NDVI, it is unlikely that this trend was purely caused by large-scale geographic factors. The results from this study suggest that increased neighborhood privilege is associated with growth in that neighborhood’s greenspace, a pattern possibly explained through increased rates of park construction and tree planting programs.

These findings point to a consistent association between high-privileged neighborhoods and increased greenspace growth. While they cannot prove a causal relationship, multiple sociological mechanisms can support them. It has been widely established that higher ICE neighborhoods have significantly more greenspace than lower ICE neighborhoods even when controlling for housing tenure and population density. Areas with increased greenspace have significantly higher property values than those with less neighborhood greenspace, potentially excluding lower-income individuals from entering those areas (Conway et al. 2010). Higher-income homeowners or landlords may be more able to shoulder the non-trivial costs of purchasing and maintaining trees for private property (McPherson 1994). The correlation between income and educational attainment has been long established (Rowntree 1902), and so it is reasonable to expect that high-ICE neighborhoods will have reasonably high educational attainment. We also confirmed this with our own sensitivity analysis (Table S2). This gives these neighborhoods a tool that may increase their effectiveness in advocating with city government for more trees or other greenspace initiatives.

While the EPA and other governmental bodies can’t regulate all aspects of urban greenspace—for instance, private lawns contribute to the greenspace of a neighborhood but are maintained by private homeowners—it can promote tree planting initiatives, encourage the development of new public parks, and provide funding for green road verges. It is not surprising that higher income neighborhoods have greater availability of urban greenspace, given the increased control that wealthy homeowners have over the vegetation on their property. Because of this, we argue that cities should invest more resources into increasing greenspace in lower and lower-middle class neighborhoods. This will help account for the disproportionate growth in greenspace in higher-privileged census tracts. It is possible that to achieve true greenspace equity, people in the lowest ICE quintile should have a higher amount of public green space compared to people in the highest ICE quintile to counteract the inequity in the quality of privately owned green spaces.

While the highest ICE quintile neighborhoods had the largest increase in NDVI, areas in the bottom 20% also experienced significant improvements to their greenspace over the study period. Most notably, they tended to do better than census tracts in the “middle class”—neighborhoods in the 20th to 60^th^ percentile. This trend can be visualized in Fig. 1, and deviates from the idea that increased neighborhood privilege will always lead to greater greenspace growth.

A census tract with multiple parks, minimal impervious surface area, and plentiful street trees may find it difficult to continue improving their green space access. Essential roads, buildings, and parking lots place a cap on the amount of greenspace a neighborhood can have. A neighborhood that lacks substantial greenspace may find it easier to make the same magnitude of greenspace change than a census tract with already plentiful parks and trees. Street trees can be planted, and vacant lots can even be converted into small parks. Along this line of reasoning, the presence of city greenspace initiatives, especially those targeting minority, lower income neighborhoods, may explain part of the increase among the “E” ranked census tracts.

However, the census tracts of the lower-middle class are lagging behind in greenspace growth. The 20–40th percentile ICE census tracts performed significantly worse than any other quintile. Despite possessing dramatically less greenspace on average than the most privileged census tracts, these tracts experienced 33% less of an NDVI increase. The lower performance of these neighborhoods follows other research suggesting the decline of the U.S. urban middle class through increased social polarization (Foster and Wolfson 2010) and white flight to the suburbs (Kye 2018). These middle-class census tracts may be neglected as cities focus on improving greenspace and social services for their least fortunate populations

Inequities in access to green space can be regarded as an example of how the Fundamental Cause Theory hypothesizes how a higher social position is linked with an array of resources and better outcomes even when health outcomes or desirable resources change. This accumulation of risk factors forms the foundation for Link and Phelan’s conception of Fundamental Cause Theory (FCT). Now a cornerstone of sociological theory, FCT states that social position should be considered a “fundamental” cause of health outcomes because it remains associated with health even when the intervening mechanisms change (Link and Phelan 1995). As long as there is a disparity in the access to resources that avoid disease, Fundamental Cause Theory states that a socioeconomic health gradient will exist. Policies that have a moderate effect on multiple diseases, or those that explicitly address inequality in fundamental resources, will have a more positive long-run impact than those that target intervening resources. Given urban greenspace’s wide-reaching impacts on human health and well-being, we posit that it is a “fundamental resource” worth addressing.

Urban gentrification can be a consequence of urban renewal and greenspace improvements (Jelks et al. 2021). However, we did not find any evidence of gentrification in this study. If gentrification were a significant factor in the observed greenspace trends, we might observe that a census tract’s change in NDVI is positively correlated with a change in ICE. However, we cannot directly observe this using ACS data since census tracts are redrawn every 10 years. The redrawing of census tracts in 2010 makes it extremely difficult to accurately compute a census tract’s change in ICE between 2001 and 2019. However, this calculation is possible for the years 2010 – 2019 for which ACS data is available and census tracts remain constant. We modeled the relationship between a census tract’s change in NDVI and change in ICE and found a small negative association (Table S3). This suggests that the effects of gentrification from greenspace are likely minimal if they exist.

### Study findings in context.

In a similar study assessing the distribution of greenspace across U.S. urban areas, Casey et al. examined the change in U.S. greenspace 2001–2010 (Casey et al. 2017). Using mid-July and yearly averaged NDVI as their objective measures, they focused on census tracts in U.S. Metropolitan Statistical Areas (MSAs) with a population above 100,000 residents. Their study focused on race-based disparities, finding that census tracts with a higher percentage of white residents tended to have significantly more greenspace in the first place and experienced a small, statistically significant increase in greenspace over the study period. On the other hand, tracts with a higher percentage of Hispanic residents had less greenspace to begin with and experienced a small decrease in greenspace from 2001 to 2010. However, their models found comparatively weaker effects when comparing percentages of Black/African-American or Asian/Pacific Islander residents in a census tract.

Our research refines the study area to focus on more densely populated urban zones, examining all tracts inside city boundaries with greater than 100,000 residents. While MSAs are more standardized than city boundaries, they also tend to overestimate the size of an urban area due to their definition on a county level (Oliveira et al. 2014; Ribeiro et al. 2019). Focusing on city boundaries allows for an examination of more ‘true’ urban areas, in the heart of the densely populated core, whereas MSAs would include census tracts from adjacent rural areas with potentially different relationships between greenspace and income.

Furthermore, a doubling of the study period to eighteen years allows for a more accurate estimation of city evolution. Improvements to city greenspace are unlikely to happen rapidly, with the construction of new parks a large-scale, expensive process that spans multiple years. For example, an initiative to increase urban greenspace in California, known as the “Urban Greening Program”, was signed into law in 2016. The program grew out of a bill originally signed in 2006, and grant recipients included projects that had been seeking funds for many years before that. The grant report for the Del Amo Community Park, in Carson, CA states that the park’s construction had been a “community priority for over two decades” and references multiple delays that blocked its road to funding and completion (Coastal Conservancy 2017). While the Del Amo Park may have experienced a longer timeline than other city greenery projects, its delays and complications reflect a reality of government funding where grants are not continuously or reliably available. Examining city greenspace change over ten or fewer years—as done in prior research (Casey et al. 2017)—allows for a snapshot of city evolution but not the full process where ideas are sparked, grants are applied for, and projects come to fruition.

Compounding the gradual process of park construction, the effects and benefits of new trees may not be noticeable until 15–20 years after planting. This is especially the case with city tree planting programs, where trees are often planted at a young age. Assuming a 20-year maturity window, a 20-year study period would allow trees planted in the early 2000s to have reached a state of more significant shade and maturity by the end of the study period. The urban tree growth model UrbTree also used a 20-year tree growth window as the foundation of its model (Kramer and Oldengarm 2010). This more long-term view of greenspace evolution allows for a greater chance of capturing the impact of efforts to improve urban greenspace. If a city initiated a tree planting program to combat a lack of urban greenery in the early 2000s, the data used in this study could detect a higher NDVI in the 2019 datapoint due to the growth of more mature trees.

### Study strengths and limitations.

This research presents an in-depth, holistic examination of greenspace growth across U.S. urban areas. By refining the study area to focus on urban census tracts and expanding the study period to nineteen years, this paper provides a needed update on U.S. greenspace equity. Nonetheless, while able to identify an association between privilege and a larger improvement in neighborhood greenspace, this study cannot make any causal claims about the fundamental drivers of greenspace growth. Greenspace may not depend on privilege and instead have a gentrifying effect, attracting people of higher income levels to live in a neighborhood. Municipal and state policymaking, as well as neighborhood social capital and civic capacity, may also impact the prioritization of greenspace development.

The choice of NDVI as a greenspace metric allows for a relatively straightforward data-collection process, but is unable to capture the quality of the greenspace in a neighborhood. Nardone et al. posits that NDVI measurements in cities with arid climates may not accurately reflect the proximity to natural environments. Past literature also suggests that the quality of greenspace can influence the strength of its positive influence on communities (Wood et al. 2018; Zhang et al. 2017). Quality metrics for greenspace include measures of biodiversity and availability of parks. Combining satellite-acquired data on greenspace prevalence with ground-level measurements on quality of greenspace maintenance is a challenging but important area for future research.

NDVI is also prone to measurement error, especially when significant cloud cover obscures an area of interest. While selecting four data points over the course of a year (January 1st, April 7th, July 12th, and September 30th) smooths some of this measurement error, data collection remains a potentially significant form of bias.

Furthermore, the definition of the study area using municipal boundaries will inevitably exclude some surrounding urban neighborhoods that we might wish to count. Municipal boundaries are often decided politically, rather than reflecting meaningful geographical differences. While discussed more extensively in the methods section, we made this choice in order to conservatively exclude more “rural” census tracts. Future research could expand this study area to more surrounding urban areas, such as the City Clustering Algorithm (Oliveira et al. 2014).

### Future directions.

Studies tracking greenspace growth serve as important accountability measures for cities as they attempt to create a more equitable urban landscape. Research efforts are needed to continue monitoring urban greenspace development, as well as developing better metrics to assess an area’s greenspace quality. Investigations into the sociological explanations behind observed greenspace trends may help city leaders most effectively target limited resources.

The U.S. is an appealing area to study urban greenspace because of its robust population statistics down to the census tract level. However, greenspace inequities extend far beyond the U.S.(Hoffimann et al. 2017; Hwang et al. 2020; Schüle et al. 2019) Further research is needed to understand the dynamics of greenspace growth outside the United States, particularly in fast-growing megacities such as Jakarta, Indonesia and Mumbai, India.

## Conclusion

Greenspace distribution remains highly stratified, and these inequities have expanded over the last two decades. However, the non-linearities of this greenspace growth suggest complicated underlying dynamics. The study provides further evidence of the struggles of the American lower-middle class, where the lag in greenspace improvements finds parallels in larger macroeconomic trends.

Such studies are important for urban planners and city government officials in creating more equitable urban landscapes that align with the ideas of environmental justice. By ensuring that neighborhoods have equal access to greenery, and equal resources to improve it, cities can weaken the fundamental link between social position and health. These measures will only become more important as cities become increasingly crowded and hot with the effects of urbanization and global warming. But harnessing the many positive impacts of greenspace can create stronger, healthier neighborhoods for all.

## Supplementary Material

Supplemental table

Supplemental table 1

**Supplementary information** The online version contains supplementary material available at https://doi.org/10.1057/s41599–023-02115-w.

## Figures and Tables

**Table 1:** A Spearman’s correlation table.

Predictor	Estimate	95% Confidence interval	*P* value

(Intercept)	0.0636	(0.0479, 0.0792)	5.43E-14
ICE	0.0198	(0.0119, 0.0277)	8.57E-07
Ecoregion: Great Plains	0.0253	(0.000721, 0.0501)	0.0462
Ecoregion: Marine West Coast Forests	−0.0285	(−0.088, 0.0319)	0.360
Ecoregion: Mediterranean California	−0.0657	(−0.0915, −0.0401)	9.38E-07
Ecoregion: North American Deserts	−0.0615	(−0.0946, −0.0285)	0.000327
Ecoregion: Northwestern Forested Mountains	0.0070	(−0.166, 0.18)	0.938
Ecoregion: Tropical Wet Forests	0.0453	(−0.0163, 0.107)	0.154
Population Density (scaled)	−0.0023	(−0.00602, 0.00146)	0.233

The estimated sample mean and standard deviation for each variable is summarized in the table, as well as the correlation of each variable to July 2010 NDVI and July NDVI change 2001–2019.

**Table 2:** The output of a linear mixed-effects model.

Predictor	Estimate	95% Confidence interval	*P* value

(Intercept)	0.07854	(0.062, 0.0951)	<2E-16
ICE Quintile E (lowest)	−0.01628	(−0.0244, −0.00818)	8.21E–05
ICE Quintile D	−0.02454	(−0.0326, −0.0165)	2.10E–09
ICE Quintile C	−0.01963	(−0.0276, −0.0116)	1.61E–06
ICE Quintile B	−0.01269	(−0.0207, −0.00471)	0.00182
Ecoregion: Great Plains	0.02527	(0.000695, 0.0501)	0.0465
Ecoregion: Marine West Coast Forests	−0.02761	(−0.088, 0.0328)	0.375
Ecoregion: Mediterranean California	−0.06469	(−0.0905, −0.0391)	1.39E–06
Ecoregion: North American Deserts	−0.06121	(−0.0944, −0.0282)	0.000352
Ecoregion: Northwestern Forested Mountains	0.00654	(−0.166, 0.18)	0.941
Ecoregion: Tropical Wet Forests	0.04643	(−0.0153, 0.108)	0.145
Population Density (scaled)	−0.00239	(−0.00615, 0.00136)	0.213

Models the percent change in July NDVI from 2001 to 2019 with ICE Quintile “A,” the most privileged ICE quintile, as the reference group. *P* values were calculated using the *lmerTest* package and Satterthwaite’s approximation.

**Table 3:** The output of a linear mixed-effects model, with ICE as a continuous variable.

Predictor	Estimate	95% Confidence interval	*P* value

(Intercept)	0.0636	(0.0479, 0.0792)	5.43E–14
ICE	0.0198	(0.0119, 0.0277)	8.57E–07
Ecoregion: Great Plains	0.0253	(0.000721, 0.0501)	0.0462
Ecoregion: Marine West Coast Forests	−0.0285	(−0.0888, 0.0319)	0.360
Ecoregion: Mediterranean California	−0.0657	(−0.0915, −0.0401)	9.38E–07
Ecoregion: North American Deserts	−0.0615	(−0.0946, −0.0285)	0.000327
Ecoregion: Northwestern Forested Mountains	0.0070	(−0.166, 0.18)	0.938
Ecoregion: Tropical Wet Forests	0.0453	(−0.0163, 0.107)	0.154
Population Density (scaled)	−0.0023	(−0.00602, 0.00146)	0.233

The coefficient estimate for “ICE” represents the expected change in percent greenspace growth from July 2001 to July 2019 with a one-unit increase in ICE. *P* values were calculated using the *lmerTest* package and Satterthwaite’s approximation.

## Data Availability

NDVI data can be found through this link (https://appeears.earthdatacloud.nasa.gov/), and demographic statistics from the U.S. Census API at data.census.gov. The cleaned data that support the findings of this study, as well as data analysis scripts, are available from the corresponding author upon reasonable request.
