# Supplementary material for: Changes in the relationship between Index of Concentration at the Extremes and U.S. urban greenspace: a longitudinal analysis from 2001–2019
Source: Humanit Soc Sci Commun. Author manuscript; Available in PMC 2026 Jan 30. (PMC12853361; doi:10.1057/s41599-023-02115-w)
Supplement: Supplemental table [file NIHMS2105179-supplement-Supplemental_table.docx]

**Supplementary Data and Figures**

| **Predictor** | **Estimate** | **95% CI** | **P-value** |
| --- | --- | --- | --- |
| (Intercept) | 0.0783 | (0.0618, 0.0949) | < 1 x 10^-16^ |
| ICE Quintile B-E (lower) | -0.0182 | (-0.0246, -0.0118) | 2.39E-08 |
| Ecoregion: Great Plains | 0.0254 | (0.000798, 0.0502) | 0.04556 |
| Ecoregion: Marine West Coast Forests | -0.0274 | (-0.0877, 0.033) | 0.37924 |
| Ecoregion: Mediterranean California | -0.0646 | (-0.0904, -0.0389) | 1.44E-06 |
| Ecoregion: North American Deserts | -0.0610 | (-0.0941, -0.0279)) | 0.00037 |
| Ecoregion: Northwestern Forested Mountains | 0.0062 | (-0.167, 0.179) | 0.94419 |
| Ecoregion: Tropical Wet Forests | 0.0461 | (-0.0156, 0.108) | 0.14751 |
| Population Density (scaled) | -0.0028 | (-0.00649, 0.000894) | 0.13851 |

Table S1: The output of a linear mixed-effects model, dichotomizing ICE between the highest quintile (A, reference) and the lower four ICE quintiles (B-E). P-values were calculated using the *lmerTest* package and using Satterthwaite’s approximation.

| **Predictor** | **Estimate** | **95% CI** | **P-value** |
| --- | --- | --- | --- |
| (Intercept) | -0.3696 | (-0.391, -0.348) | < 1 x 10^-16^ |
| Fraction >= Bachelor’s | 1.353 | (1.34, 1.37) | < 1 x 10^-16^ |

Table S2: The results of a linear mixed-effects model to describe the relationship between neighborhood educational attainment and ICE. Model fit using random effects at the city level, with the coefficient of “Fraction >= Bachelor’s” reflecting the expected increase in neighborhood ICE when the fraction of residents with at least a bachelor’s degree in a neighborhood increases by one unit. This model demonstrates an unsurprisingly significant and strong relationship between these two demographic variables.

| **Predictor** | **Estimate** | **95% CI** | **P-value** |
| --- | --- | --- | --- |
| (Intercept) | 0.02988 | (0.0255, 0.0342) | 2.526E-33 |
| **ICE Change 2010-2019** | **-0.003918** | **(-0.00697, -0.000867)** | **0.0119** |
| Ecoregion: Great Plains | -0.02578 | (-0.0321, -0.0195) | 4.127E-15 |
| Ecoregion: Marine West Coast Forests | -0.02532 | (-0.0428, -0.00785) | 0.005205 |
| Ecoregion: Mediterranean California | -0.02942 | (-0.0362, -0.0226) | 2.47E-16 |
| Ecoregion: North American Deserts | -0.0276 | (-0.0362, -0.0191) | 5.816E-10 |
| Ecoregion: Northwestern Forested Mountains | -0.02026 | (-0.0708, 0.0303) | 0.4363 |
| Ecoregion: Tropical Wet Forests | -0.001142 | (-0.0188, 0.0165) | 0.9 |
| Population Density (scaled) | -0.0006875 | (-0.00145, 7.61e-05) | 0.07764 |

Table S3: The output from a linear mixed-effects model to examine possible greenspace-driven gentrification by regressing the change in 2010-2019 NDVI on the ICE Change 2010-2019. The small negative coefficient value, as well as the relatively large p-value, suggests that the effects of gentrification are not significant in this study.
