# Supplementary material for: Changes in the relationship between Index of Concentration at the Extremes and U.S. urban greenspace: a longitudinal analysis from 2001–2019
Source: Humanit Soc Sci Commun. Author manuscript; Available in PMC 2026 Jan 30. (PMC12853361; doi:10.1057/s41599-023-02115-w)
Supplement: Supplemental table 1 [file NIHMS2105179-supplement-Supplemental_table_1.pdf]

Changes in the relationship between index of concentration of extremes and U.S. urban greenspace: a longitudinal analysis from 2001-2019

Kitch, JC[a], Nguyen, TT[b], Nguyen, QC[b], Hswen, Y[c]

[a]Department of Statistics, Harvard University, USA
[b]Department of Epidemiology and Biostatistics, University of Maryland School of Public Health, USA
[c]Department of Epidemiology and Biostatistics, Bakar Computational Health Sciences Institute, University of California San Francisco, USA

*** Corresponding author: James Celi Kitch, jckitch@alumni.harvard.edu

Supplementary Data and Figures

Predictor	Estimate	95% CI	P-value
(Intercept)	0.0783	(0.0618, 0.0949)	$< 1 \times 10^{-16}$
ICE Quintile B-E (lower)	-0.0182	(-0.0246, -0.0118)	2.39E-08
Ecoregion: Great Plains	0.0254	(0.000798, 0.0502)	0.04556
Ecoregion: Marine West Coast Forests	-0.0274	(-0.0877, 0.033)	0.37924
Ecoregion: Mediterranean California	-0.0646	(-0.0904, -0.0389)	1.44E-06
Ecoregion: North American Deserts	-0.0610	(-0.0941, -0.0279))	0.00037
Ecoregion: Northwestern Forested Mountains	0.0062	(-0.167, 0.179)	0.94419
Ecoregion: Tropical Wet Forests	0.0461	(-0.0156, 0.108)	0.14751
Population Density (scaled)	-0.0028	(-0.00649, 0.000894)	0.13851

Table S1: The output of a linear mixed-effects model, dichotomizing ICE between the highest quintile (A, reference) and the lower four ICE quintiles (B-E). P-values were calculated using the *lmerTest* package and using Satterthwaite’s approximation.

Predictor	Estimate	95% CI	P-value
(Intercept)	-0.3696	(-0.391, -0.348)	$< 1 \times 10^{-16}$
Fraction >= Bachelor's	1.353	(1.34, 1.37)	$< 1 \times 10^{-16}$

Table S2: The results of a linear mixed-effects model to describe the relationship between neighborhood educational attainment and ICE. Model fit using random effects at the city level, with the coefficient of "Fraction >= Bachelor's" reflecting the expected increase in neighborhood ICE when the fraction of residents with at least a bachelor's degree in a neighborhood increases by one unit. This model demonstrates an unsurprisingly significant and strong relationship between these two demographic variables.

**Predictor**	**Estimate**	**95% CI**	**P-value**
(Intercept)	0.02988	(0.0255, 0.0342)	2.526E-33
**ICE Change 2010-2019**	**-0.003918**	**(-0.00697, -0.000867)**	**0.0119**
Ecoregion: Great Plains	-0.02578	(-0.0321, -0.0195)	4.127E-15
Ecoregion: Marine West Coast Forests	-0.02532	(-0.0428, -0.00785)	0.005205
Ecoregion: Mediterranean California	-0.02942	(-0.0362, -0.0226)	2.47E-16
Ecoregion: North American Deserts	-0.0276	(-0.0362, -0.0191)	5.816E-10
Ecoregion: Northwestern Forested Mountains	-0.02026	(-0.0708, 0.0303)	0.4363
Ecoregion: Tropical Wet Forests	-0.001142	(-0.0188, 0.0165)	0.9
Population Density (scaled)	-0.0006875	(-0.00145, 7.61e-05)	0.07764

Table S3: The output from a linear mixed-effects model to examine possible greenspace-driven gentrification by regressing the change in 2010-2019 NDVI on the ICE Change 2010-2019. The small negative coefficient value, as well as the relatively large p-value, suggests that the effects of gentrification are not significant in this study.
